# Expression of concern: Influence of chemical composition on the amount of second phases precipitates and transformation temperatures of TiNiPdCu shape memory alloys prepared through novel powder metallurgy route

**DOI:** 10.1039/d3ra90122j

**Published:** 2023-12-11

**Authors:** Abid Hussain, Afzal Khan, Muhammad Imran Khan, Saif Ur Rehman

**Affiliations:** a Department of Mechanical Engineering, University of Engineering and Technology Peshawar Pakistan abidhussain@uetpeshawar.edu.pk; b Ghulam Ishaq Khan (GIK) Institute of Science, Engineering and Technology District Swabi, Topi 23640 Khyber Pakhtonhwa Pakistan; c Institute of Industrial Control System Rawalpindi Pakistan

## Abstract

Expression of concern for ‘Influence of chemical composition on the amount of second phases precipitates and transformation temperatures of TiNiPdCu shape memory alloys prepared through novel powder metallurgy route’ by Abid Hussain *et al.*, RSC *Adv.*, 2023, **13**, 29376–29392, https://doi.org/10.1039/D3RA05513B.

The Royal Society of Chemistry is publishing this expression of concern in order to alert readers that concerns have been raised regarding the reliability of the SEM images in Fig. 1, 3, 4, 5, 6 and 7. An investigation is underway, and an Expression of concern will continue to be associated with the article until a final outcome is reached.

In addition, the name of the second author was spelt incorrectly in the original article, and this notice provides the correct spelling.

Laura Fisher

5^th^ December 2023

Executive Editor, *RSC Advances*

## Supplementary Material

